# Burn Time of Metal Nanoparticles

**DOI:** 10.3390/ma12091368

**Published:** 2019-04-26

**Authors:** Igor Altman

**Affiliations:** Combustion Science and Propulsion Research Branch, Naval Air Warfare Center Weapons Division, 1 Administrative Circle, China Lake, CA 93555, USA; igor.altman@navy.mil; Tel.: +1-760-939-4669

**Keywords:** nanoparticle combustion, burn time, energy accommodation coefficient

## Abstract

This article will discuss the combustion of metal nanoparticles and explain the burn time dependence on particle size. In contrary to common belief in the power law (*t_b_*~*d^0.3^*), which, in our knowledge, is simply an experimental fit to data, we propose the logarithmic law (*t_b_~ln(d)*) that describes well the known results on nano-aluminum combustion. We derived the logarithmic dependence from a simple model taking into account the energy balance on the surface of a burning metal nanoparticle. The model in question is based on the small energy accommodation coefficient (EAC), which was recently utilized to solve experimental puzzles such as the significant temperature gap between the burning nanoparticle and the environment. A discussion on EAC, which value is important for the correct modeling of nanoparticle combustion, is also included. A way to generalize the considered combustion model is suggested.

## 1. Introduction

The high reactivity of metal nanoparticles makes them one of the most interesting subjects in combustion research. Engineering applications of metal nanoparticle combustion requires a deep understanding and correct modeling of the process. In particular, the burn time should be well described. This means at least both, the dependence of the burn time (*t_b_*) on nanoparticle size (*d*) and the time magnitude itself need to be well described. Many years of studies have produced unquestionable experimental results on burn times, however, there is still no theory capable of consistently explaining what has been observed.

In order to highlight the existing puzzles [1], we begin by studying the observed burn time dependence on particle size [2,3,4]. It is well known that relatively large particles (100 µm range) burn following the *t_b_*~*d*^2^ law (diffusion-limited combustion), while the kinetic-limited combustion (*t_b_*~*d*) takes place for smaller particles (10 µm range). Further reduction in particle size (<1 µm) yields the burn times, which the community tries to fit using the power law with the exponent as a fitting parameter. As a result, the *t_b_*~*d*^0.3^ dependence is commonly considered [4]. However, the main issue with this is that the exponent value of 0.3 has no physical meaning and has not been derived from a consistent model. It is worth noting that if the nanoparticle burn time dependence on particle size does axiomatically follow an integer power law, this dependence should be *t_b_*~*d*^0^, i.e., the next term in the series *t_b_*~*d*^2^, *t_b_*~*d*. Additionally, since the logarithmic law *t_b_*~*ln*(*d*) is formally an equivalent of the power law with the zero exponent, then the “zero exponent” does not mean the burn time does not depend on particle size. The latter remark is just a demonstration that the logarithmic dependence of the nanoparticle burn time on particle size that we derived based on the consistent energy balance, lies in a row with the power laws considered for larger particles. This remark does not require further discussion. At the same time the logarithmic law, if proven, eliminates the need to explain the exponent value of 0.3.

Another puzzle relating to nanoparticle combustion is the observed temperature gap between burning aluminum nanoparticles and the environment during the detected burn time of the order of 100 µs [1], while conventional heat transfer models allow for that gap during times shorter than 1 µs only. The authors were able to solve this puzzle using the small energy accommodation coefficient (EAC) in their heat balance model. Note that this small EAC was theoretically predicted and experimentally demonstrated in previous work [5,6], and it was successfully utilized for the modeling of the nanoparticle growth in different systems [7,8,9].

The current paper is an attempt to consistently examine the particle energy exchange with environment during combustion and justify the major effects that determine the burn time. We show that the burn time dependence can be obtained based on the heat exchange itself without the explicit consideration of the mass exchange. At the same time, the paper is not a comprehensive theory of the nanoparticle combustion, i.e., it does explain the experiment, but is not intended to predict it. 

Due to the importance of the small EAC for grounding our conclusions, in this paper we address issues relating to the EAC measurement as well.

## 2. Model Description

First, an assumption is made that the oxygen concentration is high enough, so that the particle temperature can reach the metal boiling point at the given ambient pressure. It is obvious that if the particle temperature cannot exceed the metal boiling point then, the particle temperature remains equal to the metal boiling point. The energy balance on the surface of this nanoparticle can be written as:

(1) $$-Q\cdot A\left(D\right)\cdot dD\;=\;\left[\varepsilon_{0}\left(D\right)\cdot \sigma T_{boil}^{4}+k_{f}\cdot \left(T_{boil}-\;T_{a}\right)\right]\cdot A\left(D\right)\cdot dt,$$

where *Q* (in Joule per m^3^ of reacting aluminum) is the combustion enthalpy at combustion temperature, *A*(*D*) is the particle surface area, the nanoparticle emissivity *ε*_0_(*D*) is proportional to the current particle diameter, *D*, [10]:

(2) $$\varepsilon_{0}\left(D\right)=q\cdot D,$$

*σ* is the Stefan-Boltzmann constant, *T_boil_* is the metal boiling point, and *T_a_* is the ambient temperature.

The radiation coefficient, *q*, is defined as:

(3) $$q=\;\frac{\int_{0}^{\infty }q\left(\lambda ,T_{boil}\right)\cdot R_{B}\left(\lambda ,T_{boil}\right)d\lambda }{\sigma T_{boil}^{4}},$$

where *R_B_* is the Planck blackbody function and:

(4) $$q\left(\lambda ,T\right)=\;\frac{12\pi }{\lambda }\;\frac{\varepsilon^{\prime \prime }}{{\left(\varepsilon^{\prime }+2\right)}^{2}+{\left(\varepsilon^{\prime \prime }\right)}^{2}}$$

with the dielectric function of the particle substance *ε*(*λ,T*) = *ε*^′^(*λ,T*) + *iε*^″^(*λ,T*). 

The free-molecular conductive heat transfer coefficient, *k_f_*, can be expressed as:

(5) $$k_{f}\;=\;\frac{\alpha_{E}Pc_{g}}{8T_{a}}\cdot \frac{\gamma +1}{\gamma -1},$$

where *α_E_* is EAC, *P* is the ambient pressure, *c_g_* is the average gas molecule velocity, and *γ* is the ratio of specific heat of gas at constant pressure to its value at constant volume.

Integrating Equation (1) with Equation (2), we obtain the time, during which the size of burning particle decreases from the initial one, *d*, to zero, i.e., the burn time:

(6) $$t_{b}\left(d\right)=\;t_{0}\cdot ln\left(\frac{d}{d_{*}}+1\right),$$

with the “characteristic” nanoparticle burn time:

(7) $$t_{0}=\;\frac{Q}{q\cdot \sigma T_{boil}^{4}\;},$$

and the “characteristic” diameter:

(8) $$d_{*}=\;\frac{k_{f}\cdot \left(T_{boil}-\;T_{a}\right)}{q\cdot \sigma T_{boil}^{4}},$$

which is a boundary between the regimes of the heat transfer. Nanoparticles smaller than d_*_ lost more energy by conduction heat transfer, while for those larger than d_*_ the radiation is more effective. 

It should be noted that it is the proportionality of the nanoparticle emissivity to the diameter that led to the logarithmic law in Equation (6).

As one can see from Equation (6), if the nanoparticle size is much smaller than the “characteristic” diameter, i.e., the conduction heat transfer is prevailing, then the burn time is proportional to the particle size:

(9) $$t_{b}\left(d\right)=\;t_{0}\cdot \frac{d}{d_{*}}\ll t_{0}.$$

In the opposite case, when the “characteristic” diameter is much smaller than the nanoparticle size, i.e., the radiative heat transfer dominates, the burn time is given by the logarithmic law:

(10) $$t_{b}\left(d\right)=\;t_{0}\cdot ln\left(\frac{d}{d_{*}}\right)\;≳\;t_{0}.$$

Interestingly, an exponential dependence of the particle size on residence time, which is formally given by the same equation, Equation (10), was obtained for the nanoparticle growth during condensation [7].

## 3. Solution Properties

**Figure 1:**
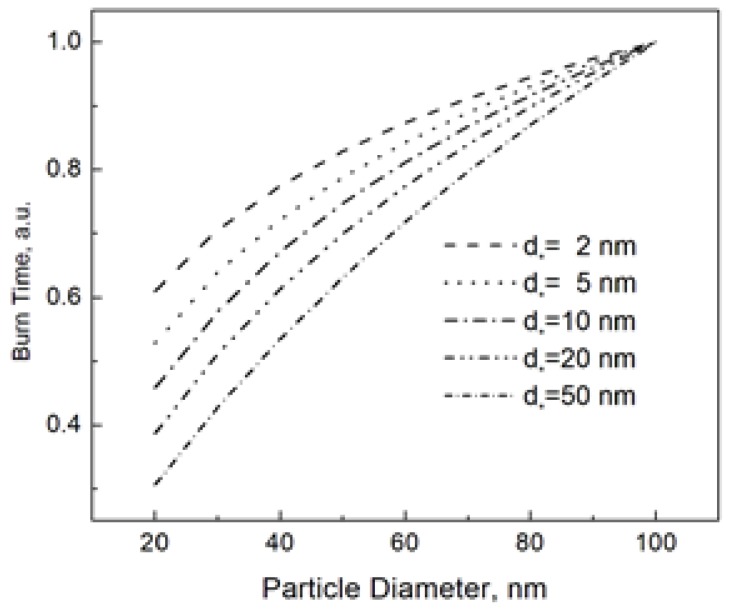
Nanoparticle burn time calculated using Equation (6) at different d_*_. The burn times at the given d_*_ are normalized to that for the nanoparticle size of 100 nm.

The “characteristic” diameter d_*_ in Equation (6) is a parameter that controls the shape of the burn time dependence. In order to illustrate this shape dependence, Figure 1 shows the burn time vs. particle size at different values of d_*_. For an easy comparison, all burn times in Figure 1 are normalized to that for the nanoparticle size of 100 nm. We claim that varying the parameter d_*_ under the logarithm in Equation (6), we can closely reproduce the power dependence *t_b_~d^n^*, which has been commonly used to describe the burn time of nanoparticles. 

**Figure 2:**
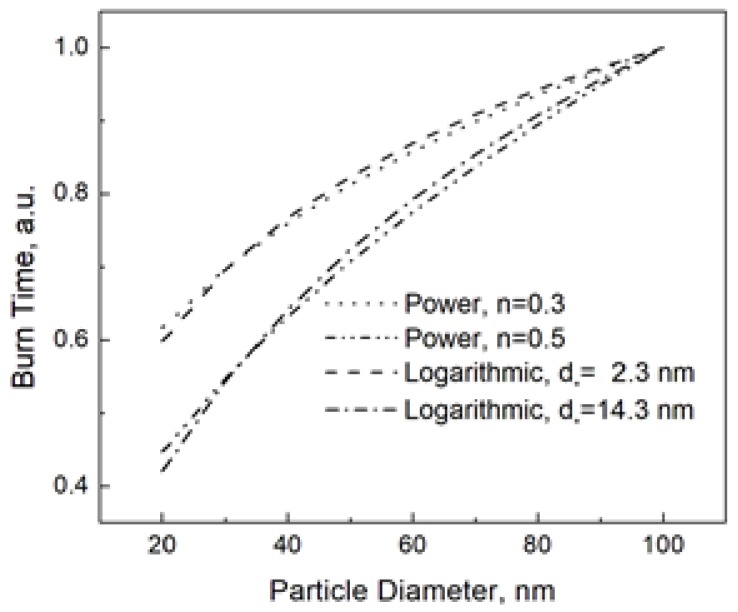
Power and logarithmic burn time laws at different parameters. The burn times at the given parameters are normalized to that for the nanoparticle size of 100 nm.

Figure 2 shows the both the power and logarithmic dependencies at *n* = 0.3 (corresponding d_*_ = 2.3 nm) and *n* = 0.5 (corresponding d_*_ = 14.3 nm). 

From Figure 2, it is understandable that the logarithmic and power dependencies are experimentally undistinguishable. Here we need to remind the reader that Equation (6) is the consequence of the energy balance, while the power dependence used to fit the experimental data has never been based on a consistent model. Then, we should rather say it in the opposite way that the power dependence reproduces the real one given by Equation (6), and, therefore, despite its irrelevance, the law *t_b_~d^n^* has usually led to acceptable fitting results.

## 4. Comparison with Experiment

**Figure 3:**
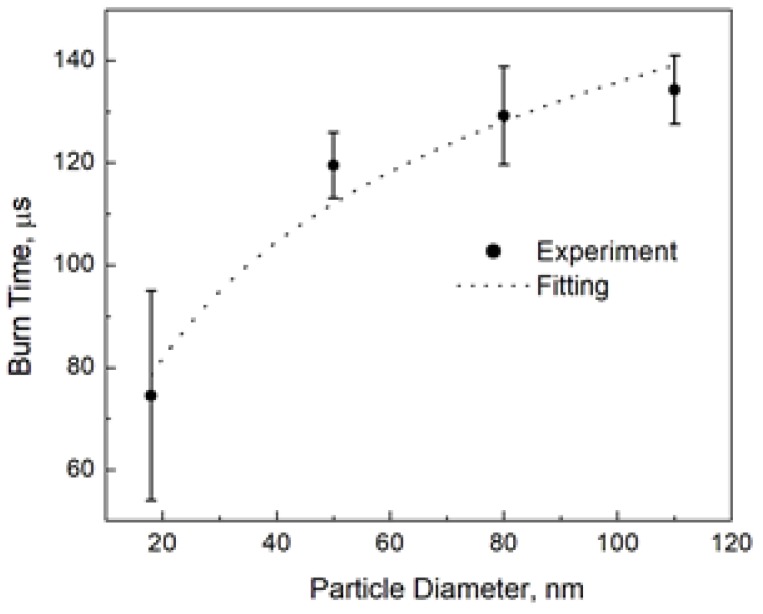
The burn time of aluminum nanoparticles [1] and fitting to the logarithmic law, Equation (6), with *t_0_* = 35 µs and d_*_ = 2.2 nm.

Allen et al. [1] reported the burn time of aluminum nanoparticles at 1500 K and 20 atm in 20% O_2_–80% N_2_. They also measured the particle temperature and found it to be about 3450 K, which corresponds to the aluminum boiling point at their pressure. We may compare the prediction of the above model with the experiment. Figure 3 shows the result of fitting the experimental burn times to the logarithmic law, Equation (6). This fitting gives *t_0_* = 35 µs and d_*_ = 2.2 nm. 

As the authors reported [1], the nominal particle sizes we used for fitting in Figure 3 are not the perfect measures. We also fitted the experimental burn times by varying the particle size and found that while the logarithmic shape of the burn time dependence remained unquestionable, the fitting parameters themselves were sensitive to different nanoparticle diameters, especially to the smallest ones. It should be also noted that the “characteristic” diameter was more sensitive than the “characteristic” time, and the range was 35–50 µs for the “characteristic” burn time and 2–7 nm for the “characteristic” diameter. However, while we used the size of solid aluminum nanoparticles for fitting in our experiment, at the combustion temperature aluminum is in the liquid state, and its density is much lower than that of the bulk material (1.65 g/cm^3^ [11] vs. 2.7 g/cm^3^). This thermal expansion leads to an increase in the “characteristic” diameter as a model parameter by about 20% compared to the fitted value but does not change the “characteristic” burn time. The liquid aluminum density should be used at the conversion of the combustion enthalpy, *Q*, from J/m^3^ to J/mol. 

The estimate of the radiation coefficient using measured values of the aluminum dielectric function [12], yields *q*~10^7^ m^−1^. Then, using *t_0_* = 35 µs to restore the combustion enthalpy from Equation (2) we get *Q*~2.8 × 10^9^ J/m^3^ ~46 kJ per mole of aluminum. This value is much less than the enthalpy of complete aluminum combustion (~800 kJ/mol), and, at the first glance, appears to be irrelevant. However, the question regarding the combustion enthalpy that is capable to sustain combustion is not as straightforward as one can expect. The enthalpy, which enters Equation (1), is the effective energy available to the burning particle, it is not the total energy released during combustion. Here we need to describe the combustion model in more detail. Although the nanoparticle temperature is equal to the aluminum boiling point, combustion cannot occur in the vapor-phase. It is because of the absence of the mechanism to sustain aluminum evaporation. Then, the aluminum oxidation occurs on the nanoparticle surface leading to the formation/existence of alumina film. The nanoparticle temperature is high enough for the suboxide evaporation. Due to the suppressed conduction heat transfer (small EAC), the energy of re-condensation of these suboxides in the particle vicinity cannot be transferred back to the particle surface. The aluminum evaporation required for the vapor-phase combustion is also impossible due to the same reason. Thus, the only energy available to sustain combustion is released in the following reactions:

(11) $$Al\left(liquid,T_{boil}\right)+\frac{m}{2n}O_{2}\left(T_{a}\right)\to \frac{1}{n}Al_{n}O_{m}\left(gas,T_{boil}\right)+\Delta H.$$

We used stoichiometric coefficients in Equation (11) in order to get the enthalpy per mole of reacting aluminum, Δ*H*. The temperature of reacting oxygen is chosen to be an ambient temperature (1500 K) that implies the negligible heat transfer between the burning particle and environment due to the small EAC. The enthalpies of reaction for different suboxides calculated using the National Institute of Standards and Technology (NIST) data [13] are presented in Table 1. Liquid alumina formation enthalpy is included in Table 1 for comparison. 

In order to estimate the effective combustion enthalpy, which determines the “characteristic” burn time in Equation (7), the comprehensive thermodynamics analysis that would provide the weights of different suboxide reactions in Equation (11) is required. The non-isothermality of the system (the temperature of the particle, on which surface the reaction occurs, is much higher than that of oxygen) significantly complicates the rigorous quantitative analysis. At the same time, some qualitative speculations are possible even without detailed consideration. 

Based on the thermodynamic data [13], we can conclude that AlO and AlO_2_ are the most preferential suboxides at the presence of condensed Al_2_O_3_ at 3450 K. The partial pressure of AlO is much higher than that of AlO_2_. However, at the ambient temperature of 1500 K, the equilibrium between these suboxides significantly shifts towards AlO_2_. Then, because the evaporation of suboxides from Al_2_O_3_, which are formed on the surface of the burning particle, occurs in the cold environment, we hypothesize that the main suboxide leaving the particle surface is AlO_2_. In this case, the combustion enthalpy is assumed to be the energy of AlO_2_ formation in Equation (11), i.e., *Q*~43 kJ/mol, which is close to the value of 46 kJ/mol restored from the fitting. We realized that this closeness is rather a coincidence, and the above qualitative thermodynamics analysis is presented to only illustrate the right order of magnitude of the combustion enthalpy we use in the model.

The value of EAC restored is of the order of 10^−4^, though it looks unexpectedly small, it does not contradict our previous results on its upper estimate of ~10^−3^ [5]. This upper estimate was obtained using the Debye temperature of solid. Taking into account that the Debye temperature of liquid is significantly lower than that of solid [14,15], we can significantly reduce the upper estimate. Further work is required to develop a model that is capable of calculating rather than estimating the value of EAC.

## 5. Remarks on EAC

As one can see, the value of EAC is important for the correct modeling of the nanoparticle heat transfer. The assumption on its small value helped to resolve a couple of experimental puzzles [1] on nano-aluminum combustion. It also allows one to derive the logarithmic dependence of the nanoparticle burn time on size discussed in this paper. At the same time, the small EAC is not widely accepted by the combustion community. We need to highlight key points relating to this issue. 

The asymptotically vanishing value of EAC at high temperatures was derived from the rigorous quantum mechanics model [5] describing energy transfer to the phonon system which distribution does define the temperature of not only solids, but that of liquids as well [16]. The small EAC value (<0.005) was demonstrated in the experiment [6]. At the same time, there is a belief that the EAC of the order of 0.1–1 claimed to be measured by the laser-induced incandescence (LII) technique [17,18] should be used instead. 

In order to reconcile these seemingly contradictive results, we need to share our current understanding of physical fundamentals of LII [19] partially discussed in Ref. [20], although a comprehensive LII analysis is, of course, beyond the scope of the current paper. The irradiated particle is an aggregate of both the system of electrons and the system of phonons. The laser pulse in LII (photon energy of 1.2 eV or greater), which cannot directly interact with phonons, excites electrons creating their non-equilibrium distribution. During the particle cooling after the laser pulse electrons are thermally isolated from phonons. The mechanism of this thermal isolation in nanostructures and corresponding experimental results could be found in Ref. [21]. 

The LII practitioners interpret the light emission from the non-equilibrium system of electrons, which are thermally isolated from phonons, as the particle thermal light emission with equilibrium emissivity. The decay of this light emission is attributed to particle cooling due to the conduction heat transfer controlled by EAC, which is further restored. Indeed, the derived value is some kind of “energy” accommodation coefficient, but it describes the energy transfer from the highly excited non-equilibrium electron system. It is unrelated to the EAC used to describe the conduction heat from a nanoparticle, where the energy transfer from the phonon system to the environment occurs. We leave a conclusion on the reliability of EAC values reported by LII to the reader. 

Our understanding of the physical fundamentals of LII has recently been supported by the experiment [22], although the authors were not able to explain what they measured. It has been found that the available laser energy was much less than that required to heat the particles up to the experimentally observed temperature. The energy deficit (the ratio of the required energy to the available one) exceeded 10. It is easy to understand that the latter result questions LII conclusions in their entirety. The explanation of the apparent paradox, however, is quite simple. The heat capacity of the system of electrons, and, therefore, the energy required to heat up electrons only, is much less than that for the particles. Then, modeling the process as the interaction of the laser with electrons at the thermal isolation of phonons does not lead to the energy deficit. It is worth reiterating that based on the energy deficit issue demonstrated [22], the conclusions made by treating all LII experiments should be completely revisited.

## 6. Discussion

The current paper deals with an oversimplified model in order to demonstrate that the suggested approach can be successfully applied for the description of the aluminum nanoparticle combustion. Now, we may discuss what can make the model more general. 

The assumption we made is that the oxygen concentration is high enough to sustain the steady state particle temperature close to the boiling point of aluminum. This assumption is based on the experimental temperature value. The boiling point enters the “characteristic” burn time, Equation (7). Since the boiling point depends on the ambient pressure, Equation (7) gives an indirect dependence of the burn time on the pressure. 

In order to get an oxidizer concentration, at which the aluminum particle can reach the boiling point, the mass transfer and reaction kinetics should be considered. Fine effects such as nitridation may also be included depending on the ambient gas composition. We can speculate, however, that, due to the suppressed conductive heat transfer (small EAC), the steady state regime is possible only at a phase transition temperature. The current paper has only considered aluminum boiling. There could be a different phase transition (melting, etc.) for other metals, and a detail analysis based on the specific metal properties is required to identify this phase transition. 

The energy balance Equation (1), which is valid for nanoparticles only, could be modified to make it applicable for a particle of an arbitrary size. Two parameters should be re-defined for that purpose, namely, the particle emissivity and the conductive heat transfer coefficient. We may suggest that:

(12) $$\varepsilon \left(D\right)=\varepsilon_{b}\cdot \left[1-exp\left(-\frac{q\cdot D}{\varepsilon_{b}}\right)\right]$$

where the bulk material emissivity, *ε_b_*, is a good approximation for the emissivity of an arbitrary particle size. The monotonous function of Equation (12) yields *ε(D)* = *q·D* for small particles, and *ε(D)* = *ε_b_* for large ones.

The conductive heat transfer coefficient can be expressed as:

(13) $$k\left(D\right)=\frac{k_{f}\cdot \frac{Nu\cdot \lambda }{D}}{k_{f}+\frac{Nu\cdot \lambda }{D}},$$

where *Nu* is the Nusselt number and *λ* is the thermal conductivity of the gas. 

For large particles, i.e., at:

(14) $$k_{f}\gg \frac{Nu\cdot \lambda }{D}\;$$

Equation (13) gives *k(D) = Nu·λ/D*. For small particles, i.e., at:

(15) $$\frac{Nu\cdot \lambda }{D}\gg k_{f}$$

from Equation (13) we obtain *k(D) = k_f_,* the coefficient used in Equation (1). 

It is obvious that Equation (15), which defines the applicability of the free-molecular heat transfer, can be re-written using the Knudsen number, *Kn*, as:

(16) $$Kn\gg \alpha_{E\;}.$$

Then, Equation (16) shows that, due to the small EAC, the free-molecular heat transfer can be formally considered for particles much larger than those satisfying the traditional condition *Kn* >> 1.

Modifying Equation (1), we can consider the general energy balance equation applicable to the burning particle of an arbitrary size as:

(17) $$-Q\cdot A\left(D\right)\cdot dD\;=\;\left[\varepsilon \left(D\right)\cdot \sigma T_{s}^{4}+k\left(D\right)\cdot \left(T_{s}-\;T_{a}\right)\right]\cdot A\left(D\right)\cdot dt,$$

where *T_s_* is the steady state temperature, which depends on the burning metal and ambient conditions, discussed above. 

As one can see, Equation (17) allows for three possible burn time dependencies *t_b_*~*ln(d_1_*), *t_b_*~*d*_2_, and *t_b_*~*(d_3_)^2^* at different initial particle sizes *d_1_* < *d_2_* < *d_3_*, exactly as that known from the experiment. A comprehensive comparison of predictions of Equation (17) with existing data on the burn times of different metals may be useful to validate the generality of our approach.

## 7. Summary

We demonstrated the possibility of the logarithmic dependence of the aluminum nanoparticle burn time on particle size. It is the direct result of the energy balance taking into account the small value of the energy accommodation coefficient (EAC). The irrelevance of the laser-induced incandescence (LII) technique to EAC measurements and the way to resolve the internal issues of LII is discussed. A possible way to generalize the considered burn time model for different metal particles of arbitrary sizes is also suggested.

## Figures and Tables

**Table 1:** The enthalpy of reaction in Equation (11) and liquid alumina formation enthalpy.

Suboxide	Enthalpy, kJ/mol
AlO	−71.6
AlO_2_	43.4
Al_2_O	95.2
Al_2_O_2_	197.7
Al_2_O_3_ (liquid)	819.1
